# Domestication as the driver of lower chronic stress levels in fish in catch-and-release recreational fisheries and aquaculture versus wild conspecifics

**DOI:** 10.1371/journal.pone.0326497

**Published:** 2025-06-25

**Authors:** Ahmad Ghazal, Richard Paul, Ali Serhan Tarkan, Irmak Kurtul, Josephine Pegg, Demetra Andreou, J. Robert Britton

**Affiliations:** 1 Department of Life and Environmental Sciences, Bournemouth University, Poole, United Kingdom; 2 Department of Ecology and Vertebrate Zoology, Faculty of Biology and Environmental Protection, University of Lodz, Lodz, Poland; 3 Department of Basic Sciences, Faculty of Fisheries, Muğla Sıtkı Koçman University, Menteşe, Muğla, Türkiye; 4 Marine and Inland Waters Sciences and Technology Department, Faculty of Fisheries, Ege University, İzmir, Türkiye; 5 South African Institute for Aquatic Biodiversity, Makhanda, South Africa; 6 Department of Ichthyology and Fisheries Science, Rhodes University, Makhanda, South Africa; CIFRI: Central Inland Fisheries Research Institute, INDIA

## Abstract

The manipulation of species’ attributes through selective breeding can produce domesticated traits including decreased stress responses (i.e., selecting for high stress resilience). Common carp *Cyprinus carpio* (“carp”) have been domesticated for centuries, with domesticated forms frequently used to enhance recreational catch-and-release fisheries around the world. In Atlantic salmon *Salmo salar* (“salmon”), two primary strains are evident, a wild strain and domesticated aquaculture strain. Here, we compared scale cortisol concentrations (a biomarker of fish chronic stress levels) between domesticated carp in catch-and-release pond fisheries and wild carp in waters with no angling. Carp of low scale cortisol concentration were apparent in all sampled populations, suggesting individuals of low stress sensitivity are encountered in both wild and domesticated strains, and in natural and captive environments. Carp with relatively high levels of scale cortisol were, however, only present in wild carp, suggesting high phenotypic variability in their chronic stress responses, with some individuals being highly sensitive to stress. In some wild carp, elevated scale cortisol concentrations could also have been indicative of adaptive responses to their heterogenous environments. We then compared wild versus farmed salmon scale cortisol levels, and found a similar pattern, with relatively high scale cortisol levels only detected in wild fish. These results indicate that while domesticated carp and salmon are exposed to potentially stressful environments, they appear to have some resilience against the adverse effects of chronic stress.

## Introduction

Domestication aims to increase the socio-economic and recreational benefits that are derived from plant and animal species [1]. Fishes are a group of vertebrates with significant domestication histories with common carp *Cyprinus carpio* (“carp”) having domesticated strains produced for approximately 8000 years [2]. Atlantic salmon *Salmo salar* (“salmon”) has been domesticated relatively recently (circa 1970s) but has undergone intense domestication for food production (farmed fish currently make up 70% of global salmon consumption) [3]. Many domesticated fish are present in the environment through regulated releases that support conservation programmes and enhance recreational fisheries, and through escapes from aquaculture sites [4,5].

The traits of domesticated fishes are artificially selected, where the intense selection has produced individuals that are, for example, faster growing, and more stress and disease resistant [6,7]. These fish are produced in captive conditions where food is not limiting, predation risk is minimised, and the rearing environment is usually benign [6]. Conversely, the adaptations and traits of wild fish represent solutions to selection pressures driven by ecological problems involving heterogeneous environments, high resource competition and relatively high predation risk, which manifest through differential fitness amongst wild phenotypes [6,8]. The result is that domesticated strains of carp differ from their wild conspecifics through higher growth rates, larger body sizes, and more varied body shapes, colours and scale patterns [4,9,10]. Domestication can also result in increased risk-taking by individual fish [11]. However, domesticated strains can re-adapt to the wild in relatively short evolutionary periods, such as across several generations [10]. In salmon, a wild strain and a domesticated aquaculture strain is evident, where the latter has reduced genetic diversity, rapid growth, lower reproductive investment and decreased stress responses compared with wild conspecifics [12,13].

Domesticated carp are frequently used to enhance recreational angling in catch-and-release fisheries (C&R; all captured fishes are released alive) [14]. Carp with strong domesticated traits are very vulnerable to capture [14,15], with their capture vulnerability further increased by anglers releasing large amounts of angling bait into the water that alter carp foraging behaviours and time budgets [16]. Indeed, this introduced bait is often consumed by individual fish in high dietary proportions [5,17]. However, C&R is an acutely stressful event [18], with short-term responses to capture including changes in blood chemistry and ‘whole-body’ stress responses [19]. Nevertheless, some individual fish are captured on multiple occasions across short time periods within C&R fisheries (e.g., over 20 times in approximately 3 months) [20], raising welfare concerns around recaptures leading to elevated levels of chronic stress [5]. Domesticated salmon are also present in the wild due to escapes, where introgression with wild salmon has altered the life history and phenological traits of wild populations, with most of these alterations being maladaptive [21].

Scale cortisol concentration is a recognised biomarker of fish chronic stress [22], with concentrations influenced by energetically intense events across periods of intermediate duration (e.g., up to 30 days) [23,24]. This is in contrast to circulating cortisol concentrations (e.g., in plasma) that only represent acute stress responses (e.g., up to 48 h). In the last decade, the development of scale cortisol as a chronic stress biomarker has resulted in it being applied to many fish species to measure how different environmental conditions affect their chronic stress levels, including carp [22,24]. In European sea bass *Dicentrarchus labrax*, experiments revealed that scale cortisol concentrations successfully differentiated between chronically stressed and non-stressed individuals across a range of stressor related contexts, whereas circulating cortisol levels (including plasma) were unable to do so [25]. Given the differences in the artificially versus naturally selected traits present between domesticated and wild fishes, the aim here was to apply scale cortisol concentration as the biomarker to identify how the domestication of carp and salmon influences their chronic stress profiles. No other cortisol biomarker was used given these tend to indicate acute responses only [25]. For carp, comparisons in scale cortisol concentrations were between domesticated fish present in C&R fisheries (where angling capture of some individuals was considered as frequent) and wild carp (sampled from water bodies where their population had been self-sustaining, producing multiple generations for at least 15 years and were not exploited by C&R angling) (Table 1). For salmon, comparisons were between domesticated fish produced in intensive cage aquaculture (where feeding is through formulated pelletised food and stock densities can be very high relative to wild salmon) versus wild fish (sampled from a river where no hatchery reared juvenile salmon had been released in recent years and where fish were reliant on natural prey resources for their food supply). We used both carp and salmon in the study to identify whether differences in scale cortisol concentrations are apparent between wild and domesticated fish more generally, rather than in one species only. However, we do not compare the scale cortisol concentrations directly between the two species, as these concentrations tend to species specific and so comparisons between species are not necessarily informative [22–25].

**Table 1:** Overview of carp *Cyprinus carpio* used in the study, including site numbers (*cf*. Fig 1), locations, size range (as mm where recorded, otherwise by mass), population age for the wild populations (as time since introduction, taken from stocking records and local knowledge), sample sizes, and scale cortisol (SC) concentrations (mean, minimum (min), maximum (max); pg mg^-1^) in both recreational catch and release (C&R) fisheries and wild populations. Exact locations for the recreational fisheries are not provided to protect business confidentiality as all were managed as private businesses. As carp in the fisheries were not self-sustaining then their population age is not provided.

Site type	Site	Country	Location	Approximate population age (years)	Size range	n	Mean SC (± 95% CI)	SC (min)	SC (max)
Recreational C&R fisheries	1	Southern England	–	–	138–508 mm	61	4.10 ± 0.48	1.07	10.06
	2	Southern England	–	–	420–560 mm	10	2.11 ± 0.56	1.12	3.67
	3	Southern England	–	–	380–680 mm	48	2.69 ± 0.50	0.72	7.19
	4	Southern England	–	–	360–615 mm	25	2.68 ± 0.67	0.85	8.69
	5	Southern England	–	–	250–560 mm	29	3.93 ± 0.97	1.19	11.55
Wild populations	6	South Africa	−30.61153, 25.54232	≈ 200 years	1–5 kg	27	17.35 ± 10.44	1.00	110.58
	7	Kenya	−0.76374, 36.35226	25 years	225–750 mm	56	18.65 ± 3.96	2.27	60.74
	8	Türkiye	40.17611, 25.86417	20 years	1–4 kg	34	30.87 ± 4.23	7.77	58.48
	9	Türkiye	39.13406, 41.71012	20 years	1–4 kg	35	10.07 ± 3.27	1.74	51.95
	10	Southern England	50.66403, −1.95956	15 years	150–775 mm	39	82.75 ± 29.26	4.11	350.00

## Materials and methods

### Scale sample collection

Carp scale samples were collected from five recreational C&R fisheries where all fish were of hatchery origin and were considered as domesticated, with no known natural recruitment. Angling pressure was high in these fisheries (multiple anglers per day, including competitions; [5]), with mandatory C&R of captured fishes (Table 1). Scales were also collected from five wild, self-sustaining carp populations which were not exploited by angling (Table 1). The recreational C&R fisheries were all ponds in southern England (2–10 ha in area, depths to 2 m), with their exact locations not provided to protect business confidentiality as each was run as a private business based on angling. Their fish communities were all dominated by carp (generally < 5 kg), but with no carp stocked for at least two years prior to sampling. The carp were sampled during fish stock assessment exercises completed with seine nets during 2023, with the captured carp measured (fork length, nearest mm), and between 3 and 5 scales collected from each fish that were then stored at room temperature in a paper envelope.

Of the five wild populations, one was also located in Southern England, at Little Sea, Dorset, where a self-sustaining population had formed since their introduction by anglers in the early 2000s (Table 1). Scale samples were collected from these fish during fish removal exercises in summer 2023 that were designed to reduce carp impacts on macrophytes of conservation importance. As there are few known other self-sustaining carp populations in England due to temperature constraints [26], the remaining four populations were sampled from reservoirs and lakes in Türkiye and South Africa during 2023, and Kenya in 2011 (Table 1). Stocking records and local knowledge indicated that these carp populations had been present in the waterbodies for up to 200 years (Table 1). Carp samples were collected by gill nets (set over-night) or seine nets, with scales collected from the captured individuals. Up to 5 scales were collected from individual carp, with these scales also stored dry in paper envelopes at room temperature. For these samples, lengths were not recorded but individual mass was, where the equivalent lengths fell within the range of those sampled in England (e.g., [27]). Ageing of individual fish from scales was not completed due to the potential for individuals in some populations to have more than one growth check formed on scales in a year (e.g., South Africa; [28]), which would have resulted in erroneous age estimates in the absence of an age validation method [29]. All carp were considered as a minimum of two years old from their body sizes.

For salmon, the scales from wild salmon were available from a river in Northwest England where adults migrating upstream were sampled at a fish pass on a weir in the lower river reach for stock assessment purposes, with the fish considered as being recent arrivals into the river from the sea. The aquaculture scales were obtained from whole fish being sold in supermarkets in Southern England, where the fish had been reared in cage aquaculture facilities in Scotland. Scale collection and storage was as described for carp.

### Scale cortisol analyses

Scale material (30−100 mg) was taken from the samples of each individual fish and the cortisol extracted. The protocol used was as developed in [30]. The protocol started with washing ten scales three times with methanol (2.5 minutes per wash), as this does not leach cortisol from the scale [22]. The scale material was then cut into small pieces, transferred into a reinforced 2 ml tube, with cortisol extraction completed by adding internal standard (Cortisol-d4) and HPLC grade methanol to make up the volume to 1 ml, followed by wet grinding the fish scales using a Fisherbrand™ Bead Mill 24 Homogenizer for 1 hour (2.8 mm metal beads were also added to aid extraction). These were then centrifuged (3 minutes), 200ul of supernatant collected and transferred into HPLC vials, with analysis on an Agilent 1290 Infinity II UHPLC system (Agilent Technology, Palo Alto, CA, USA) connected to an Agilent 6546 Quadrupole Time-of-Flight Mass Spectrometry instrument equipped with an electrospray ionization (ESI) source. The column used for compound separation was a 2.1 × 50 mm 1.8 μm C18 analytical column (Zorbax Eclipse Plus C18, Agilent), protected by a 2.1 mm × 5 mm 2.7 μm C18 guard cartridge (Agilent). For the chromatography, injection volume was 10 µL, and the mobile phases were 0.1% of formic acid in deionized water (phase A) and methanol (phase B) at a constant flow rate of 0.55 mL/min, with the gradient used being 5% B at 0 min; gradient elution changes from 5% to 55% B in 0.3 min; from 55% to 80% B in 3.7 min; from 80% to 100% B for 1 minute. The ESI source operated in negative ionization mode under conditions of nebulizer gas at 35 psi, drying gas flow rate and temperature at 12 L min^-1^ and 250 °C, respectively. The sheath gas was set at 350 °C with a flow rate of 12 L min^-1^. Capillary voltage was 2500 V, while the fixed fragmentor, skimmer, and octupole voltages were 150, 65, and 750 V respectively. Subsequently targeted MS/MS was carried out with a set precursor ion (407.2 and 411.2 m/z) using a collision energy of 20 eV. The MassHunter Quant Workstation software was used to process the data obtained by UHPLC–QTOF in targeted MS/MS mode. The analytical method was validated following UKAS ISO 17025 guidelines for quantitative analytical methods, with some exceptions. A 7-point calibration in the range of 0.1 ng mL^-1^ to 10 ng mL^-1^ was linear (r2 > 0.99), intra-day and inter-day accuracy and precision fell within acceptable criterion (accuracy between 88% and 107%, with precision at < 3.5 CV%). Assuming a 100 mg scale sample then the limit of quantification was 0.5 pg mg^-1^ and limit of detection was 1 pg mg^-1^.

### Data analyses

Descriptive data of the carp scale cortisol concentrations by fish origin were calculated, with visual inspection via box plots. Carp scale cortisol concentrations were then tested between fisheries and the wild using Gamma Generalized Linear Modelling (GLM) in the R environment (v.4.3.2) [31], using the glmmTMB package [32]. Prior to model fitting, a thorough data exploration was conducted [33]. The fixed effects were water type (fishery or the wild) and fish length, and site was the random factor (Table 1). The optimal fixed structure of the model was determined through backward selection using AIC (ΔAIC≤2). For salmon, differences in scale cortisol between wild and farmed fish were tested using permutational univariate analysis of variance (PERANOVA), with Euclidean distance and 9,999 permutations using the *adonis2* function implemented in the R package vegan [34].

### Ethical note

Sampling was completed under UK Home Office Project Licence P47216841 and following ethical approval by the Animal Welfare and Ethical Review Board of Bournemouth University.

## Results

**Fig 1:**
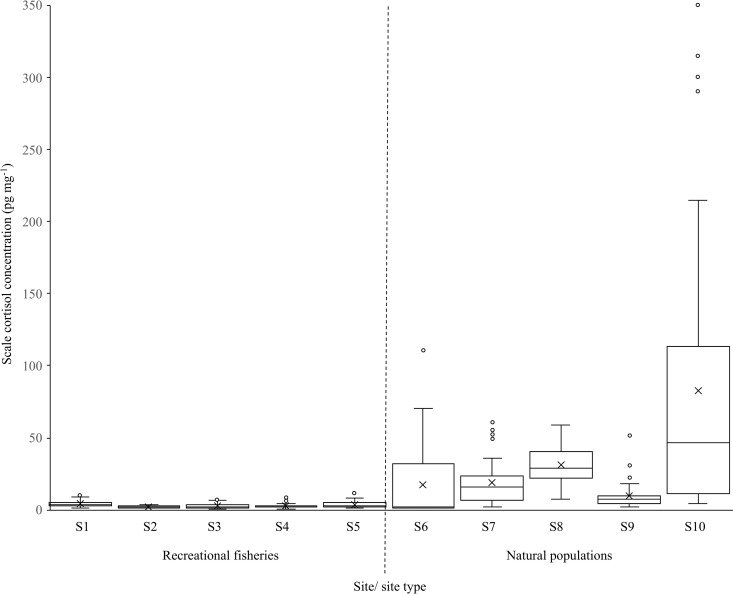
Box plot comparing the distribution of scale cortisol concentrations of carp *Cyprinus carpio* sampled from five recreational fisheries (S1 to S5; Table 1) and five wild populations (S6 to S10; Table 1), where the dashed vertical line separates the two site types. Horizontal lines represent 10, 25, 50, 75 and 90 percentiles, x is the mean and clear circles are outliers.

Carp scale cortisol concentrations across the five recreational fisheries ranged from 0.72 to 11.55 pg mg^-1^, with an overall mean concentration of 3.36 ± 0.31 pg mg^-1^ (n = 173; Table 1; Fig 1; S1 File). In the five wild populations, some scale cortisol concentrations were within the range detected in carp in the recreational fisheries, but with a substantial proportion of fish also having much higher cortisol values (range 1.00 to 350.00 pg mg^-1^; overall mean: 32.16 ± 7.31 pg mg^-1^ (n = 191; Table 1; Fig 1; S1 File). The GLM revealed these scale cortisol levels were significantly higher in wild carp versus those in the recreational fisheries (estimate: −2.75 ± 0.17, Z = −16.57, P < 0.001). The effect of fish length was not significant (estimate: 0.001 ± 0.001, Z = −1.61, P = 0.11).

**Fig 2:**
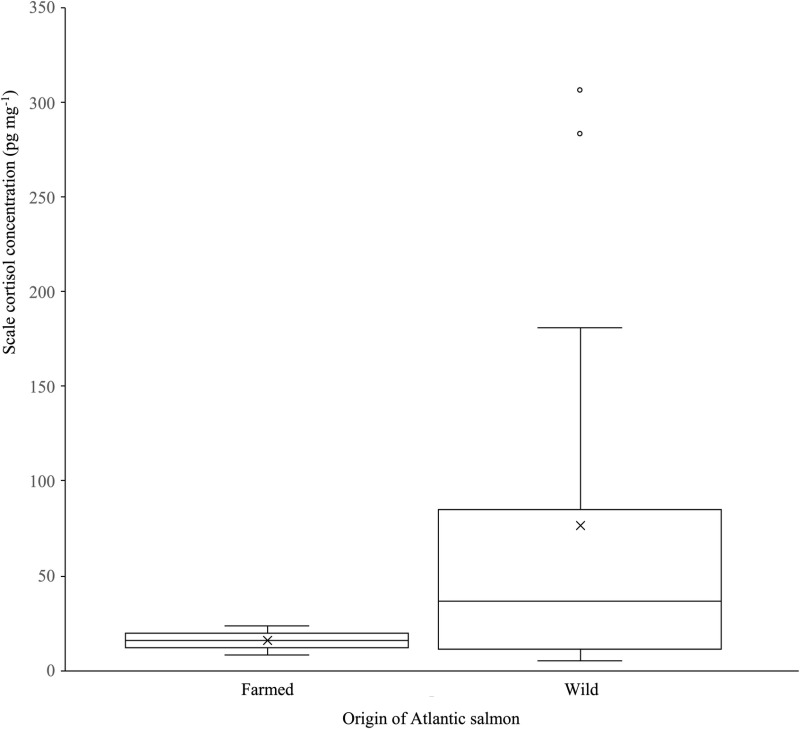
Box plot comparing the distribution of scale cortisol concentrations of Atlantic salmon produced in aquaculture (“Farmed”; n = 22) and a river in Northwest England (“Wild”; n = 16). Horizontal lines represent 10, 25, 50, 75 and 90 percentiles, x is the mean and clear circles are outliers.

For wild salmon, scale cortisol concentrations ranged from 5.46 to 306.59 pg mg^-1^, with an overall mean concentration of 76.31 ± 47.28 pg mg^-1^ (n = 16; Fig 2; S1 File). This compares to farmed salmon where scale cortisol concentrations were 8.87 to 19.93 pg mg^-1^, with an overall mean concentration of 14.55 ± 2.26 pg mg^-1^ (n = 10; Fig 2; S1 File). The farmed salmon scale cortisol levels were significantly lower than those of the wild salmon (Peranova; F = 8.696, P < 0.001; Fig 2).

## Discussion

Individual carp with relatively low values of scale cortisol were apparent in all the sites and populations sampled, irrespective of whether they were wild or domesticated. Conversely, individuals with high scale cortisol concentrations were only present in wild carp sampled from natural environments. Scale cortisol concentrations have been used successfully as a biomarker of fish chronic stress across numerous species and studies (e.g., [22–25]. Consequently, the scale cortisol concentration profiles here suggest that carp domestication has resulted in individuals of low phenotypic variability whose sensitivity to chronic stress is much reduced compared with many wild conspecifics. The low concentrations of scale cortisol in carp are despite these fish being in environments that expose them to high angling pressure where capture events would be at least acutely stressful [18,19]. Conversely, the response of wild carp to their more heterogeneous natural environments appears to have resulted in higher phenotypic variability in the sensitivity of individuals to chronic stress, resulting in their much greater ranges in scale cortisol concentrations. That this contrasting pattern in scale cortisol concentrations was also seen in domesticated (farmed) versus wild salmon provides some support for this, especially as farmed salmon have been selected to be more stress resilient than their wild conspecifics [12].

The production of carp in hatcheries over multiple generations that have been selected for specific traits attractive to anglers and fishery managers (e.g., faster growth rates, specific scale patterns, body shape; high vulnerability to angling capture; [14,15]), thus also appears to produce strains of low stress sensitivity. The salmon results also suggested that the aquaculture strain has also been selected for stress resilience, with this having a genetic basis [12,13]. Nevertheless, the low scale cortisol concentrations of the domesticated fishes might also relate to their environments. For the domesticated carp, rather than providing stressful conditions, their pond environments might instead provide more homogenous and low stress environments than those encountered by their wild conspecifics. For example, in C&R fisheries, their risk of predation is reduced through the management of piscivorous fauna (so predator presence is much reduced versus wild conditions), and the daily release of angler baits (which are often based on aquaculture feeds) ensures high food availability, with the diet of most fish present being dominated by these baits [5,17]. This predominance of angling baits in fish diets could result in high recapture rates (with some fish in C&R fisheries recaptured on multiple occasions). However, some fish are never captured [20] and species such as carp often show hook avoidance behaviours after a single capture or even through social learning [35]. Thus, these C&R fisheries potentially provide relatively benign and predictable environments for carp and, as some fish might only be captured infrequently, their exposure to multiple stressful events could be low. Salmon in aquaculture are also exposed to a very homogenous environment where food supply is high and predation risk is minimal [36], whereas wild salmon are potentially exposed to high heterogeneity in their environment, such as through patchy food resources and relatively high predation risks during foraging that, in combination, could result in some individuals expressing relatively high chronic stress levels [37]. Accordingly, there is a need to better understand the role of these managed environments and their interaction with the domesticated traits of the fish in producing the low scale cortisol levels measured.

The relatively high scale cortisol levels detected in many of the wild carp and salmon suggested these fish are more chronically stressed than their domesticated conspecifics. While this is likely to be the case for those individuals with very high levels of scale cortisol (e.g., the outliers), it might not be the case for those with only modestly elevated scale cortisol concentrations versus domesticated conspecifics. For these wild fish, it might be that rather than relatively high scale cortisol concentrations suggesting chronically stressed individuals, these concentrations were the result of how the individuals are responding in an adaptive way to their heterogeneous environment [5,38,39]. These adaptive responses could include the up regulating of cortisol in response to the demands of activities including reproduction and migration, where the changing hormonal levels ensure the provision of the energy needed to support these [39,40]. Consequently, in the wild fishes, some caution is needed in the interpretation of their elevated scale cortisol levels in relation to chronic stress levels.

The finding that the domesticated carp have consistently low scale cortisol concentrations and stress sensitivity is then potentially important for their welfare. While there remains high scientific uncertainty around topics such as fish sentience, consciousness and pain (e.g., [41,42]), it has been argued that human uses of fish should be sustainable and do no more harm than necessary [43]. Providing that domesticated, low stress phenotypes are unable to escape into the wider environment and affect local gene pools [44], the production and use of carp within C&R recreational fisheries could limit associated welfare issues. However, further work is needed in this area to understand the dynamics of stressor exposure and trait expression between wild and captive contexts. Work is also needed to explore the behavioural syndromes present in wild and domesticated fishes, including whether low stress sensitivity consistently correlates with boldness and high activity levels, and whether the consumption rates of stress resilient fish are higher than those of low stress resilience due to differences in their risk-taking during foraging [10]. In addition, understanding the interactions of stressors and stress responses that are both acute and chronic is important [45], but was beyond the scope of this study to investigate. However, understanding how the daily activities and stressor exposure affects acute stress responses, and how these build up to develop chronic stress responses, and their relationships with scale cortisol levels, remains uncertain. Notwithstanding, experiments have indicated scale cortisol levels do differentiate between chronically stressed and non-stressed individuals, whereas circulating cortisol levels cannot due to their more acute and short-term nature [25]. In addition, the salmon data have to be treated with some caution as these are limited in sample number and information on the rearing conditions and the aquaculture sites were unavailable to the study.

In summary, there were substantial differences in the scale cortisol concentrations between carp in recreational fisheries and the wild, and between Atlantic salmon produced in aquaculture and the wild, where scale cortisol is a recognised biomarker of chronic stress. We posit that for both species, the domesticated fish were selected for a suite of traits that either directly or indirectly resulted in high resilience to chronic stress, explaining their relatively low values of scale cortisol. For the wild fishes, their higher ranges of scale cortisol concentrations were considered to be driven by the different responses of individuals to their heterogenous environments, resulting in high phenotypic variability in their stress sensitivities. The elevated scale cortisol concentrations of some wild individuals versus their domesticated conspecifics might also be relate to how the wild fish adapt to their changing environments and daily needs. Nevertheless, the results here indicated that while carp in recreational fisheries and salmon in aquaculture are regularly exposed to events that could elevate their stress levels, these fish appear to have physiological traits that provide some resilience against the harmful effects of chronic stress.

## Supporting information

S1 File Raw scale cortisol data for common carp *Cyprinus carpio* and Atlantic salmon *Salmo salar* used in the study. (XLSX)
